# *Alaria alata* in Terms of Risks to Consumers’ Health

**DOI:** 10.3390/foods10071614

**Published:** 2021-07-13

**Authors:** Weronika Korpysa-Dzirba, Mirosław Różycki, Ewa Bilska-Zając, Jacek Karamon, Jacek Sroka, Aneta Bełcik, Magdalena Wasiak, Tomasz Cencek

**Affiliations:** 1 Department of Parasitology and Invasive Diseases, National Veterinary Research Institute, Partyzantow Avenue 57, 24-100 Pulawy, Poland; mrozycki@piwet.pulawy.pl (M.R.); ewa.bilska@piwet.pulawy.pl (E.B.-Z.); j.karamon@piwet.pulawy.pl (J.K.); jacek.sroka@piwet.pulawy.pl (J.S.); aneta.belcik@piwet.pulawy.pl (A.B.); tcencek@piwet.pulawy.pl (T.C.); 2 Department of Pathology, National Veterinary Research Institute, Partyzantow Avenue 57, 24-100 Pulawy, Poland; magdalena.wasiak@piwet.pulawy.pl

**Keywords:** *Alaria alata*, parasite, risk, meat, venison, pork, one health

## Abstract

*Alaria alata* flukes are cosmopolitan parasites. In Europe, the definitive hosts are red foxes (*Vulpes vulpes*), wolves (*Canis lupus*), and raccoon dogs (*Nyctereutes procyonoides*), as well as animals that belong to the Felidae family. Intermediate hosts, such as snails and frogs, are the sources of infection for definitive hosts. The developmental stages of *A. alata* mesocercariae may occur in paratenic hosts, including many species of mammals, birds, and reptiles, as well as in wild boars (*Sus scrofa*), which are important from the zoonotic point of view. Because there are no regulations concerning the detection of *A. alata* in meat, this fluke is usually detected during official obligatory *Trichinella* spp. inspections. However, a method dedicated to *A. alata* detection was developed. The growing popularity of game and organic meat has led to an increased risk of food-associated parasitic infections, including alariosis, which is caused by the mesocercarial stage of *A. alata*. The aim of this article is to highlight the problem of *A. alata* as an emerging parasite, especially in the terms of the increasing market for game and organic meats that have been processed with traditional methods, often without proper heat treatment.

## 1. Introduction

*Alaria alata* is a widespread trematode that is considered a potential cause of a human disease called alariosis, which is associated with the consumption of raw or undercooked meat of intermediate (snails, frogs) or paratenic (mainly game) hosts of this parasite.

In recent years, a growing population of wild boars (*Sus scrofa*) has been reported in Europe. However, since 2018, a decrease in the number of these animals has been observed because of African swine fever or preventive actions undertaken in the European Union against the spread of this disease [1]. Another reason for this decrease is the greater popularity of hunting in Europe and the greater interest in venison products. The meat of wild boars is low in fat, has a high content of wholesome protein, and has a higher level of iron in comparison with pork, poultry, and beef. In addition, it has a very low level of cholesterol and a high content of unsaturated fatty acids, vitamins (thiamine and riboflavin), and minerals (sodium and potassium) [2]. Thus, it is considered an element of a healthy diet. This has resulted in an increase in the consumption of game meat as well as risks related to food-associated parasitic infections, including those caused by *Alaria alata* [3,4]. The *A. alata* species is mainly found in Europe, whereas on other continents, there are other species of this trematode, such as *A. americana*, *A. mustelae*, *A. intermedia,* and *A. marcianae* [5,6,7]. Epidemiological studies have shown that this parasite is widespread in various environments because of its broad range of possible paratenic hosts, including many species of birds, amphibians, reptiles, and mammals [8,9,10,11]. However, a key role in the life cycle of *A. alata* is played by snails, frogs, and tadpoles, which is why the aquatic environment is the most conducive for the spread of this parasite [12]. Wójcik et al. (2002) showed that the percentage of snails and frogs infected by *A. alata* larvae depends on the season, ranging from 30% in autumn to 100% in spring. This availability of snails and frogs infected with *A. alata* makes them an important source of infection for definitive and paratenic hosts. Because of their feeding habitats, wild boars are considered the main source of *A. alata* for humans [13,14]. Wild boars play a much greater role in the spread of *A. alata* than farm animals, such as pigs. However, the growing popularity of free-range animal farming raises the possibility of the infection of domestic pigs that are kept outdoors [11,15]. There is consumer demand for ecological products as well as those manufactured using traditional and natural methods; nevertheless, in recent decades, the amount of ecological production of meat in the European Union has remained at 3% of the total meat production. In 2017, about 5% of cattle, 6% of sheep and goat, and 3% of poultry production were classified as organic, whereas in the entire pig farming sector, ecological farming accounted for less than 1% of total production. Still, the ecological production of pigs and poultry shows an upward trend [15]. A questionnaire completed by participants in proficiency tests concerning *Trichinella* spp. organized by the National Veterinary Research Institute in Poland revealed that the mesocercarial stage of *A. alata*, which is known as *Distomum musculorum suis* (DMS) (a term that is currently rarely used), was occasionally found during examinations of the meat of pigs and wild boars for *Trichinella* spp. (unpublished data). This situation is probably similar in other countries, where the reference method for *Trichinella* spp. detection is used. Until recently, the presence of mesocercariae in meat was considered only a quality defect with no threat to human health. However, by consuming raw or undercooked venison, pork, frog legs, or snails containing larval forms of *A. alata*, humans become paratenic hosts and may suffer from the symptoms of alariosis [10]. Sporadic reports of alariosis cases have resulted in the classification of this disease as an emerging disease [4]. The reports of alariosis in humans came mainly from the United States and Canada and were related to the American *Alaria* species. Nevertheless, it is undeniable that *A. alata* also has a pathogenic potential and may cause alariosis after the consumption of raw or semi-raw traditional and homemade products made from meat infected by mesocercariae [4]. The symptoms of this disease are usually not specific, and therefore, its diagnosis is very difficult. There are no public safety regulations for diagnosing meat containing *A. alata*, except for a statement in Regulation (EU) 2017/625 of the European Parliament and the Council that indicates that game meat in which parasites are found should be considered as unfit for consumption. Because there is no official method for the detection of *A. alata*, this trematode is mainly detected during official Trichinella inspections that use the artificial digestion method [16,17]. The aim of this article is to draw attention to the problem of the presence of *A. alata* in food and the emergence of new risks that have not yet been considered, such as the presence of this parasite in the meat of wild boars and pigs from organic farms as well as in meat products that are processed without proper heat treatment.

## 2. Characteristics of *A. alata*

**Figure 1:**
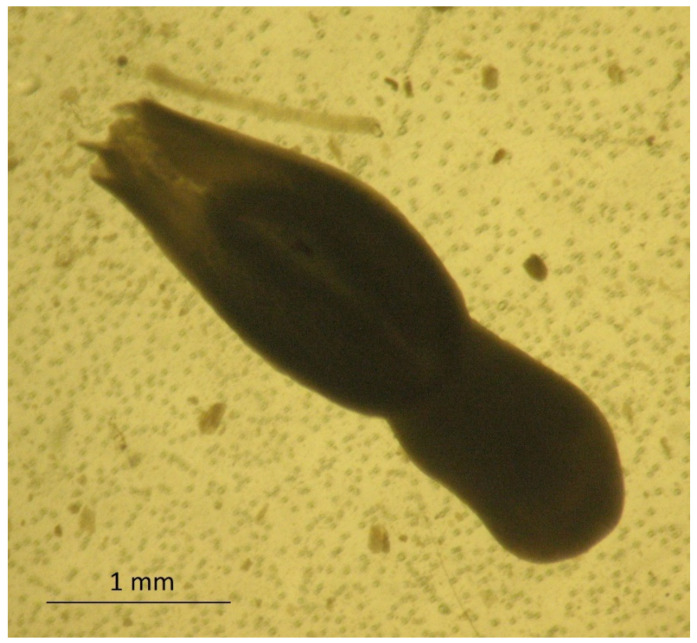
The adult stage of *A. alata* isolated from the small intestine of a red fox; 40× magnification (by J. Karamon).

The adult stage of *A. alata* was described in 1782 by Goeze, whereas the larval stage of this parasite was revealed by Gestaldi in 1854. Further descriptions of *A. alata* were made by Duncker, who studied the larval stages of these trematodes in swine muscles [18,19,20,21,22]. In 1842, Bugge identified a link between the presence of mesocercariae in frogs and pigs [23]. The relationship between *A. alata* and its mesocercarial larval stage, DMS, was demonstrated in 1953 [24]. *Alaria alata* belongs to the family *Diplostomatidae* and the genus *Alaria*. The species of this parasite inhabit different regions. *Alaria alata* is common in Europe, while *A. mustelae*, *A. intermedia*, *A. marcianae*, *A. arisaemoides*, *A. canis,* and *A. taxideae* can be found in North and South America [25,26,27,28,29,30]. The body of *A. alata* in the adult stage is 3 to 6 mm long and 1 to 2 mm wide, and it is divided into two sections. The front part of this fluke has a wing-like shape and ends in an additional clinging Brandes organ. It contains four clavate cells, which despite their glandular appearance do not have ducts [29]. However, according to recent research by Nacheva and Manikovskaya (2019), the Brandes organ is a morphofunctional unit that performs a primary function in the digestion of food by means of developed glandular structures, and it specializes in secretory activity [31]. The rear part of *A. alata* has a cylindrical shape and contains most of the internal organs (Figure 1).

**Figure 2:**
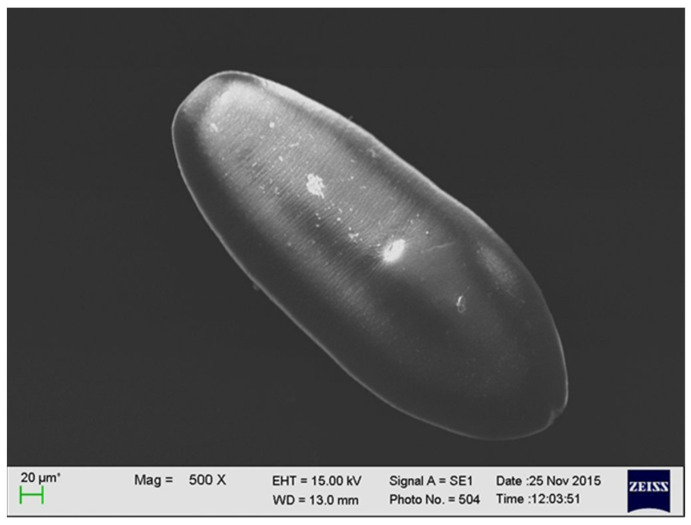
The larval stage of *A. alata* detected in the muscle tissue of a wild boar (by M. Wasiak).

The larval stage of *A. alata* has the shape of an oval, reaches up to 0.5 mm in length, and has fine parallel lines. It is equipped with a mouth and abdominal sucker [32] (Figure 2). In several studies, a series of electron microscopy photos of larval forms originating from wild boars revealed additional sucker-like surface structures.

## 3. Life Cycle of *A. alata*

**Figure 3:**
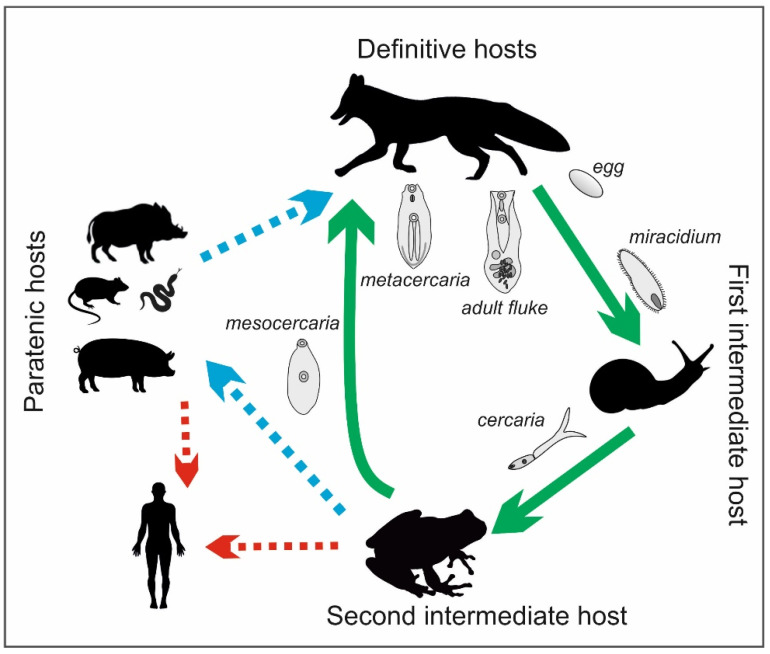
Life cycle of *A. alata* (by J. Karamon).

**Figure 4:**
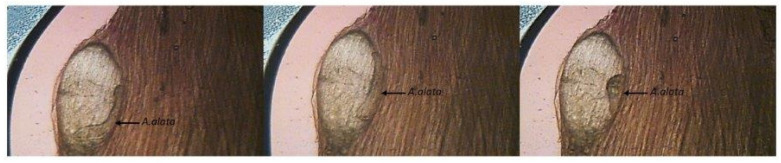
*A. alata* movement sequence in a muscular cyst of a wild boar (by M. Różycki) (the movie showing movement of *A.alata* is included in the Supplementary Materials Video S1).

The life cycle of the *Alaria* genus is complex and involves definitive, intermediate, and paratenic hosts (Figure 3). The definitive hosts are carnivores, including foxes, wolves, raccoons, lynxes, martens, badgers, dogs, and cats [11,33,34]. They become infected by eating frogs or tadpoles that contain mesocercariae, whose length can reach up to 0.5 mm (Figure 4). These parasites migrate through abdominal and thoracic cavities or via the circulation to the lungs, where the mesocercariae enter the metacercarial stage. Next, they are swallowed and develop in the small intestine, reaching 3–6 mm in length and 1–2 mm in breadth as adult flukes [32]. In studies on the distribution of the parasites in the small intestines of foxes, *A. alata* were detected mostly in the anterior parts of the intestines in almost all infected foxes (99.4%) [35]. Eggs (oval, size 98–125 × 62–81, light brown and operculated with a lid–operculum), which are a dispersive form of the parasite, are laid in definitive hosts and excreted with the feces into the environment. Miracidia, an invasive form of the parasite in intermediate hosts, are released from the eggs in aquatic environments, and they infect the first intermediate hosts, which are freshwater snails (e.g., *Helisoma*, *Planorbis* spp.). The miracidia develop into sporocysts that hatch a fast-moving larval stage—cercariae—which leave the snails, penetrate tadpoles or frogs, and develop into non-reproductive mesocercariae. The consumption of these amphibians by carnivores completes the life cycle of *A. alata*. However, as mentioned before, the life cycle of this parasite can also involve paratenic hosts, such as wild boars, mice, rats, martens, polecats, and pigs, as well as wild birds and some species of snakes and lizards. Similarly to the definitive hosts, they can be infected by eating mesocercariae from intermediate (tadpoles or frogs) or other paratenic hosts [12,33,34]. Within these hosts, the mesocercariae do not reach the stage of adult flukes; however, they can survive for months in the connective tissues between muscles or the adipose tissue, which constitute a kind of reservoir of this fluke for definitive hosts or other paratenic hosts. The migration of the mesocercariae from one paratenic host to another does not reduce the infectivity of the parasite [32]. In addition, humans can become paratenic hosts by consuming mesocercariae that are present in raw or undercooked game, pork, frog legs, or snails [10]. Infections with *A. alata* in humans cause alariosis.

## 4. Detection of *A. alata* in Definitive and Paratenic Hosts

The presence of adult flukes in definitive hosts is mainly connected with the microscopic detection of eggs in fecal samples or post-mortem parasite recovery from intestinal content. However, the diagnosis of *A. alata* infection in the meat of paratenic hosts is performed during inspections for *Trichinella* spp. Therefore, most reports of the presence of *A. alata* in wild boar meat in the literature come from tests for the presence of *Trichinella* spp. using the reference magnetic stirrer method with artificial digestion (MSM) according to ISO 18743 [17]. This technique is not dedicated to the detection of flukes of *Alaria* spp. In 2006, the German Federal Institute for Risk Assessment pointed out the existence of a risk of infection with alariosis for game consumers [36]. At that time, because of the lack of an appropriate method for detecting this parasite, the real number of infected wild boars could not be determined. Therefore, in 2010, the mesocercaria migration technique (AMT) was developed for the detection of *A. alata*. In the AMT, a sample of minced meat weighing 30 ± 2 g is transferred to a strainer, which is placed in a funnel and immersed in warm water (46–48 °C). After 90 min, 20 mL of the liquid is drained into a measuring cylinder and then into a Petri dish or larval counting basin, and it is viewed under a stereomicroscope or trichinoscope at a magnification of 15–20× [32]. Subsequently, German researchers used the method described above to examine archived wild boar meat samples that were classified as not containing *A. alata* mesocercariae during the detection of *Trichinella* spp. with the artificial digestion method. The AMT showed that 11.5% of the samples tested contained *A. alata* mesocercariae [10]. The limited possibility of detecting *A. alata* mesocercariae using artificial digestion was caused by their lower resistance to the digestive fluid (HCL/pepsin), which damaged the parasites and caused the loss of their characteristic mobility, making their identification difficult. Due to their characteristic shape and size, *A. alata* mesocercariae often do not reach the final stage of the MSM (examination of sediments) because they stay in the strainer. Therefore, one of the reasons for the low detection of mesocercariae with the MSM may also be the inadequate diameter of the mesh size in the sieves used for the tests [37]. However, according to ISO 18743:2015, which has been indicated as a reference method in the EU Commission Regulation 2020/1478 since October 2020, the diameter of the mesh size may vary from 180 to 200 µm. A larger mesh size may provide a greater possibility for the detection of *A. alaria* mesocercariae. Moreover, these parasites are most often located in the layer of connective tissue between the muscles, especially where there is a large amount of adipose tissue; however, for MSM testing for trichinosis, it is advised that samples are free of fat and fascia [38]. As a result, in official statistics, the presence of *A. alata* might be underreported. Studies were also conducted in Latvia to determine the level of potentially false-negative results found using the MSM compared with the level in the results obtained using the AMT. In these analyses, it was found that 40% of the samples tested with the MSM contained *A. alata* mesocercariae, but after using the AMT, this percentage increased to 76.7%. In addition, a significant difference in the number of mesocercariae detected was observed. Their number per gram ranged from 0.02 to 1.22 when using the AMT and from 0.02 to 0.56 when using the MSM. In 13 (21.7%) samples, mesocercariae were found only on the sieve during the ASM test. This number indicates that these samples were false negatives when tested with the MSM [37]. In studies performed in the Czech Republic between 2012 and 2013 with the AMT, the percentage of wild boar meat samples containing *A. alata* mesocercariae was 6.8%; however, when using the MSM, no mesocercariae were found in any of the 221 samples tested [39]. In Lithuania, during routine tests for the detection of *Trichinella* spp., the percentage of simultaneously detected samples containing *A. alata* mesocercariae was 7% [37]. In Poland, veterinary inspectors observed the presence of *A. alata* during regulatory testing of wild boar carcasses for trichinellosis. A questionnaire was sent to participants in proficiency tests for *Trichinella* spp. detection in Poland in 2020 (489 participants), which revealed the occurrence of mesocercariae in wild boar meat samples at the level of 4%. According to the data obtained, *A. alata* were found only in wild boars. However, according to data collected by the Veterinary Research Institute in Poland, 33% of the wild boars inhabiting the southeastern region of Poland (which is rich in water reservoirs) were infected with mesocercariae (unpublished data).

## 5. *A. alata* in Red Foxes in Europe

Recently, an increase in the population of red foxes has been observed in Europe [40,41,42,43,44]. These animals increasingly approach human neighborhoods (in rural and urban areas) in search of food. Such close contact with foxes may result in the dispersion of invasive eggs into environments that pets and farm animals inhabit; therefore, it may represent a threat to human health [45]. Studies conducted in Spain confirmed that the infection of foxes with *A. alata* is more common among wild animals that live near moist areas; this fluke was present in foxes that lived near rivers and was absent in foxes that inhabited the desert regions of southern Spain [46]. In addition, in Poland, the percentage of red foxes infected with *A*. *alata* varies significantly among different regions, as it depends on the presence of surface water. It was found that these differences ranged from 15.2% in the southern areas of Poland to 90% in the North [47]. A similar percentage (96%) of foxes infected with *A. alata* was observed in Lithuania, a country bordering Poland to the northeast [48]. Tylkowska et al. (2018) observed that 54.7% of foxes from northwestern Poland were infected with *A. alata*, especially those living near water reservoirs [45]. By comparing these results with those of previous studies in the southern region of Poland, where the percentages of foxes with *A. alata* were 39.2% and 31.6%, it can be concluded that there has been an increase in infections in this region [49]. An increase in the population of infected foxes was observed in Denmark—from 10.9% in 1984 to 16.9% in 2014 [50,51]. Reports from Italy and Croatia showed that the numbers of red foxes with *A. alata* were low—5.3% and 4.7%, respectively [52,53]. In some European countries, including Great Britain and Greece, these trematodes were not found [54,55]. Generally, in Europe, the percentage of foxes infected by *A. alata* varies, with a very high or moderate to low prevalence of this trematode. The prevalence of *A. alata* in red foxes in Europe is summarized in Table 1.

## 6. *A. alata* in Wild Boars in Europe

In recent years, in some European countries, there has been an upward trend in the occurrence of *A. alata* mesocercariae in game animals [3,10,39,56]. The infection of wild boars with this fluke depends on the boars’ exposure to sources of these parasites, and it is correlated with the number of foxes that are definitive hosts living in the same territory. By consuming parasitized snails, amphibians, or other paratenic or intermediate hosts that carry mesocercariae, wild boars become secondary paratenic hosts [32]. The larvae migrate from the intestinal lumen through the intestinal wall into the muscle, fat, and glandular tissues [57]. The appearance of *A. alata* mesocercariae in cysts in wild boars’ muscle tissues is presented in Figure 4.

In France, between 2007 and 2011, 27,582 samples of wild boar meat received from 502 hunting districts were tested using the MSM, and a relatively low rate (0.6%) of *A. alata* mesocercariae infection was found. The positive samples came from 12% of the hunting districts, indicating that the presence of this parasite was not restricted to a specific area and that there was a risk of further spread [3]. From 2007 to 2014, the French National Reference Laboratory for Foodborne Parasites conducted a survey that showed that the strongest circulation of this fluke in wild boars occurred near the rivers in the northeastern and central regions of France [58]. According to French regulations, testing for *Trichinella* spp. in wild boars intended for personal consumption is voluntary; thus, the real prevalence might be underestimated. An evaluation of data on the prevalence of *A. alata* in wild boars in Germany showed that 0.02% to 3.06% of them were infected. However, these results were based on the artificial digestion method; therefore, to estimate the real percentage of infected animals, the results mentioned above should be recalculated with a magnitude that is two to three times greater, resulting in a prevalence of 12% to 15% [10]. Studies carried out in Austria revealed that 4.3% of wild boars were infected with *A. alata* mesocercariae; among them, 86.4% of the positive samples were collected from the southern part of the country [57]. Results from 2013 to 2014, in which 6% of 348 wild boar meat samples that were tested using the AMT contained *A. alata* mesocercariae, confirmed the relatively low average presence of this parasite in Austria. Considering the previous results, it was found that this parasite is enzootic in the floodplains along the Danube river and in the area bordering the Czech Republic, where studies by Paulsen et al. (2013) revealed that 6.8% of the wild boars were infected with *A. alata* [39,59]. In Poland, Strokowska et al. (2020) examined 221 meat samples from wild boars that were hunted in five provinces localized in the northeastern part of the country in order to detect *A. alata* using the AMT. The presence of mesocercariae was confirmed in 98 (44.3%) carcasses. Such a high percentage of positive samples was probably due to the fact that in the area where the wild boars were found, there are many lakes, rivers, and swamps, which constitute an ideal living environment for intermediate hosts of this fluke [13]. In the same year, Bilska-Zając et al. (2020) published the results of research on the presence of *A. alata* in wild boars in the remaining 12 provinces in Poland. As part of this work, 3589 samples were tested with the AMT, and 151 (4.2%) were found to be positive. The highest incidence of infections—60% and 50%—was found in two regions in northern Poland, while in samples from other voivodeships, the percentages were much lower (0–15.1%). The differences in the obtained results were most likely related to the different environmental conditions in the individual regions [4]. In other parts of Europe, current data on the occurrence of this parasite are limited [14]. The prevalence of *A. alata* in wild boars in Europe is summarized in Table 2.

## 7. The Pathogenicity of *A. alata*

The pathogenicity of *A. alata* is correlated with the intensity of infections caused by high levels of intake of mesocercariae. Studies conducted on experimentally infected primates revealed that repetitive intake of mesocercariae causes an increase in the number of eosinophils in the blood or tissues (eosinophilia) and an increase in serum immunoglobulin E (IgE), which may lead to a general anaphylactic reaction with symptoms of tachycardia, a drop in blood pressure until vasomotor collapse, and unconsciousness [32]. The migration of mesocercariae causes a polyphasic change in the muscles and subcutaneous lesions in paratenic hosts, such as wild boars. Studies conducted on European minks showed that this is indicated by the infiltration of mononuclear cells and the appearance of granulomatous tissues at different maturation stages, leading to muscular and subcutaneous fibroplasia. The inflammation most likely results from direct tissue damage rather than an immune reaction targeted toward the parasitic antigens [60]. The flukes are generally considered to be nonpathogenic for definitive hosts; however, intensive infections can be responsible for enteritis [61]. To date, the sources of human alariosis cases were frog legs and goose meat [62,63,64]. The symptoms of this disease range from low-grade respiratory and cutaneous symptoms to diffuse unilateral subacute neuroretinitis (DUSN) and anaphylactic shock, with possibly lethal consequences [32,62,63,65,66]. The pathogenicity of *A. alata* has been poorly studied because the symptoms described above were observed in humans infected with other species of *Alaria* spp.

## 8. *A. alata* as a Potential Threat in the Production of Food of Animal Origin and Preventive Actions

Humans may act as paratenic hosts; in some countries, depending on local dietary habits, they can be infected by eating frogs (frog legs) or any predators of frogs, among which the wild boar is the main source of infection [67]. Frog-eating birds (herons, birds of prey, etc.) must also be taken into account as a source of human infection, even though these are not very popular dishes and are not normally consumed. There are also other sources of infection, but they are highly unlikely; these include Mustelidae (badgers, weasels, otters, etc.), Procyonidae (raccoons and coatis), which have been found to harbor the mesocercarial stage in their tissues, and even reptiles [9,14,68]. The human hazards related to the consumption of meat products that contain mesocercariae of *A. alata* depend on various factors, such as prior freezing of the meat, the amount of meat consumed, and the methods used in the processing of the meat [57]. Freezing is recommended for inactivating many parasites, including *Trichinella* or *Toxoplasma* [69]. Gonzales-Fuentes et al. (2015) pointed out that freezing game meat to an internal temperature of at most −13.7 °C inactivates mesocercariae [70]. The survival of the larvae of *A. alata* at the temperatures in refrigerators (4 to 8 °C) is very high, even during long-term storage; therefore, the potential risk for consumers remains high [58]. To date, there is no precise information about the doses required for infections. However, after analyzing confirmed alariosis cases, it can be assumed that the severity of the symptoms is correlated with the number of larvae taken in [62,64,65,71]. According to current knowledge, heat treatment is the most effective method for the inactivation of *A. alata* mesocercariae in wild boar meat. Heating at 72 °C for 2 min kills mesocercariae; therefore, the meat becomes fit for consumption [72]. In the work of Portier et al. (2011), it was shown that *A. alata* larvae could survive for at least five days when frozen (−18 °C) [3]. The most effective method for killing these flukes—as in the case of *Trichinella* spp.—is cooking at 71 °C [38]. In addition, hygienic production is very important for minimizing the risks to consumers, as smear infections can occur during meat processing [62,64,66,71]. It is difficult to estimate the risk linked with the consumption of raw or undercooked meat products made from organic or free-range pigs. Studies conducted in Serbia on diaphragm samples collected from 72 free-range pigs showed that the percentage of samples infected with *A. alata* mesocercariae was 2.77%. The researchers then underlined that the risk of human alariosis increases in regions where there is a tradition of making homemade pork products [73]. In addition, in other countries, delicacies made from raw ground pork are very popular dishes. Among them, there are types of fresh sausages, such as Italian sausage, bratwurst, Polish steak tartare, and German *mett*. These products are made from chopped, ground, or even pureed uncooked pork meat. In some territories, such as France and some Nordic countries, the consumption of game meat is related to the historical culture, in which this type of meat is shared among hunters and their families. Therefore, this group of consumers is especially exposed to the consumption of meat infected by *A. alata* [36,58]. In 2014, in Germany, studies were performed to determine the survival rate of *A. alata* larvae during the production of raw cured meat products, such as raw ham, salami, and raw sausage. These studies intended to clarify whether mesocercariae are eliminated during the production of these products and if traditional meat products play a role as sources of *A. alata* infections in humans. In the experiment, the meats of wild boars and raccoons that were positive for the presence of *A. alata* were used. A comparison of the three different technological processes showed that no live larvae were found in any of the ready-made hams, which proved that 100% of *A. alata* mesocercariae were inactivated during production. However, 11.9% of salami sausages and 18.2% of the second type of raw sausage contained mesocercariae 24 h after preparation in the initial fermentation stage. Therefore, even tasting the meat during production may lead to an intake of *A. alata* mesocercariae. These results indicate that the consumption of raw sausages in particular may be risky for consumers, especially if these products are consumed immediately after production [74]. The German Federal Institute for Risk Assessment (BfR) conducted an evaluation to determine the risk of infection with parasites after consumption of game meat. This type of product is generally consumed in low amounts in Germany (200 to 400 g per person each year). However, the consumption of game meat in Germany has increased in recent years, and a certain group of people, including hunters, their relatives, and their friends, can consume 50–90 times more meals containing game each year [75,76,77]. There is also an increasing interest in medium or rare game meat, which is pink at the core. The document mentioned above includes a recommendation to thoroughly cook game meat, raw game sausages, and raw meat products before consumption [72].

## 9. Conclusions

In 2003, the Swiss Federal Office for the Environment classified *A. alata* as a parasite in Risk Group 2 with zoonotic potential. It is now considered a potential foodborne parasite [72]. Despite the usually mild symptoms of *A. alata* infections, these parasites may be dangerous to both animals and humans. People who consume raw or semi-raw products made from wild boars and free-range pigs should be aware of the potential risks caused by *A. alata* [10]. In order to fully estimate the risks associated with the consumption of homemade meat products, further research considering different types of meat processing methods is needed [78]. However, *A. alata,* as well as other food-borne parasites, can be inactivated in food products by using proper heat treatment and production hygiene. This review on *Alaria* and *alariosis* contributes to the concept of One Health, harmonizing various compounds and practices that act together to facilitate human health, animal health, and the environment. The article aimed to raise awareness of health management for the disease. To achieve the goal of One Health, we have to bring together the human and animal health sectors and identify the complex epidemiology of the disease. This review provides support to develop effective policies to prevent and manage public health, animal health, and agricultural sectors. The same approaches were indicated by other authors, such as Shamsi (2019), who underline food safety as a global concern that cannot be viewed in isolation and as a matter of importance for all countries [79].

## Figures and Tables

**Table 1:** Prevalence of *A. alata* in red foxes in Europe.

Country	Foxes Infected by *A. alata* (%)	Analysis Method	References
Poland	15.2-0	SCT ^1^	[47]
Lithuania	96	SCT ^1^	[48]
Denmark	16.9	IST ^2^	[51]
Italy	5.3	SCT ^1^	[52]
Croatia	4.7	Microscopic examination of intestinal content	[53]
Greece	0	Sieving and sedimentation	[54]
Great Britain	0	Scraping and sedimentation	[55]

^1^ Sedimentation and counting technique; ^2^ intestinal scraping technique.

**Table 2:** Prevalence of *A. alata* in wild boars in Europe.

Country	Wild Boars Infected by *A. alata* (%)	Analysis Method	References
France	0.6	MSM ^1^	[3]
Germany	0.02–3.06	MSM ^1^	[10]
Austria	4.3–6	AMT ^2^	[57,58]
Czech Republic	6.8	AMT ^2^	[39]
Poland (northeast)	44.3	AMT ^2^	[13]
Poland (except northeast)	4.2	AMT ^2^	[4]

^1^ Magnetic stirrer method; ^2^ mesocercariae migration technique.

## Supplementary Materials

The following is available online at https://www.mdpi.com/article/10.3390/foods10071614/s1, Video S1: The movements of *A. alata* larvae present in a muscular cyst.
